# The Role of the PGC1*α* Gly482Ser Polymorphism in Weight Gain due to Intensive Diabetes Therapy

**DOI:** 10.1155/2009/649286

**Published:** 2009-04-07

**Authors:** Samir S. Deeb, John D. Brunzell

**Affiliations:** ^1^Division of Medical Genetics, Department of Medicine, University of Washington, Seattle, WA 98195-7720, USA; ^2^Division of Metabolism, Endocrinology, and Nutrition, Department of Medicine, University of Washington, Seattle, WA 98195-7720, USA

## Abstract

The Diabetes Control and Complications Trial (DCCT) involved intensive diabetes therapy of subjects with type 1 diabetes mellitus (T1DM) for an average period of 6.5 years. A subset of these subjects gained excessive weight. We tested for association of polymorphisms in 8 candidate genes with the above trait. We found the Gly482Ser polymorphism in the peroxisome proliferator-activated receptor *γ* coactivator-1*α* (PGC1*α*) to be significantly associated with weight gain in males (*P* = .0045) but not in females. The Ser allele was associated with greater weight gain than the Gly allele (*P* = .005). Subjects with a family history of type 2 diabetes mellitus (T2DM) were more common among those who gained excessive weight. We conclude that T2DM and the Gly482Ser polymorphism in PGC1*α* contribute to the effect of intensive diabetes therapy on weight gain in males with T1DM.

## 1. Introduction

The major complications of T1DM are microvascular disease leading to visual impairment and nephropathy, as well as premature cardiovascular disease [1]. The DCCT was designed as a randomized prospective trial to evaluate the benefits of intensive diabetes therapy compared with conventional therapy [2]. After a mean of 6.5 years of intensive diabetes therapy there was a reduction in retinopathy, nephropathy, and neuropathy. Two negative outcomes of intensive diabetes therapy were a threefold increase of episodes of severe hypoglycemia [2] and marked weight gain in a subset of individuals [3]. However, it was shown that the increase in incidence of severe hypoglycemia did not account for excessive weight gain [4]. The top quartile (Q4) of increase in body mass index (BMI) in the intensive therapy had a mean weight gain of 17 kg and their average BMI increased from 24 to 31 [3]. Concomitant with their excess weight gain, they developed components of the metabolic syndrome, including central obesity and insulin resistance.

The aim of this study was to identify genetic factors that contribute to excess weight among T1D patients upon intensive insulin therapy. We tested for association of this trait with sequence variants in 8 candidate genes that are implicated in obesity and/or T2DM (Table 1). The DCCT study offers a unique opportunity to find such genes since it is a large and very well characterized prospective study of subjects with T1DM that have been exposed to intensive therapy and experienced well-documented changes in weight. Whereas there are many pathways to central obesity, the weight gain due to intensive diabetes therapy in this well-characterized DCCT cohort, represents a homogeneous phenotype in which to search for central obesity genes.

## 2. Methods

### 2.1. Subjects

The DCCT was designed as a randomized prospective trial to evaluate the benefits of intensive diabetes therapy with multiple insulin injections compared with conventional therapy. A total of 1441 subjects, aged 13–39 years at baseline, 50% females and over 95% Caucasian, were randomized to conventional or intensive therapy and followed for 3.5–9 years (mean 6.5 years). Subjects in the conventional therapy group [5] typically received one or two insulin injections per day and had a quarterly follow-up at their DCCT clinic. Intensive therapy subjects practiced more rigorous diabetes management by taking three or more insulin injections per day or using an insulin infusion pump, self-monitored their blood glucose four or more times per day, and visited their DCCT care providers monthly to achieve HbA_1c_ levels at least one percentage point lower than the conventional therapy group. All enrolled subjects were in good general health.

The 582 (48% females and 52% males) subjects in this study were in the intensive therapy group and were 18 years of age or older at DCCT entry [2]. They had previously been divided into quartiles of change in BMI over the 6.2 years of the DCCT [3]. The top quartile (Q4) of increase in BMI in the intensive therapy had a mean weight gain of 17 kg and their average BMI increased from 24 to 31. Concomitant with their excess weight gain, they developed components of the metabolic syndrome, including central obesity and insulin resistance, which are strong risk factors for T2DM and for familial combined hyperlipidemia [5].

### 2.2. Selection of Candidate Genes

Previous reports of linkage and association of gene variants with obesity indices and/or T2DM were the primary criteria in prioritizing the candidate genes for this association study. In addition, priority was given to genes that contain common variants with proven functional consequences. Originally, 7 candidate genes were chosen, and more recently the *FTO* (fat and obesity associated protein) gene was added to this study because of the recent findings that polymorphisms in this gene were associated with obesity (see what follows).

The 8 candidate genes and the respective single nucleotide polymorphisms that were tested for association in this study are listed, in order of decreasing priority, in Table 1. We previously showed that the Pro12Ala polymorphism in the peroxisome proliferator-activated receptor*γ*2 (PPAR*γ*2), a transcription factor that is critical for adipogenesis, was associated with obesity and T2DM [6]. This polymorphism is among a few that have been confirmed to be associated with T2DM [7]. A second good candidate gene (*PPARGCA1*) encodes the PPR*γ* coactivator-1*α* (PGC-1*α*), common variants which, particularly the Gly428Ser substitution, were observed in some studies to be associated with obesity and T2DM [8–10]. PGC-1*α* controls the expression of a number of genes involved in energy homeostasis [11, 12] that were shown to be coordinately reduced in individuals with insulin resistance and T2DM [13, 14]. Polymorphisms in the glucocorticoid receptor (GR) gene have been reported to be associated with altered glucocorticoid sensitivity and central obesity [15–17]. 11*β*-hydroxysteroid dehydrogenase 1 (11*β*-HSD1) which plays an important role in determining tissue glucocorticoid levels has also been implicated in features of the metabolic syndrome in both humans and transgenic mice by amplifying the effects of glucocorticoid action [18–20]. The gene encoding the G protein *β*3 subunit (*GNB3*) was considered as a good candidate for the metabolic syndrome and T2DM because it contains a common polymorphism (C825T) that was observed to be associated in some studies with development of obesity in young individuals across different ethnic groups, body fat distribution and hypertension. Furthermore, the T allele causes alternate splicing with functional consequences [21–23]. A genome wide scan for the metabolic syndrome detected significant linkage to chromosomal region 3q27 where the *APM1* gene encoding adiponectin is located [24]. Furthermore, genetic variation in *APM1* was observed to be associated with obesity, insulin resistance [25], and T2DM [26]. Neuropeptide Y (NPY) is a major regulator of food consumption and energy homeostasis. Increased NPY signaling in the hypothalamus leads to obesity and its complications such as T2DM and cardiovascular disease. Polymorphisms in the *FTO* gene were shown by a genome-wide association analysis [27] to be associated with BMI and with predisposition to childhood and adult obesity (reviewed in [28]).

### 2.3. Genotyping

DNA samples from the subjects were prepared from peripheral white blood cells at the Diabetes/Endocrine Research Center at the University of Washington, USA. Single nucleotide polymorphism (SNP) genotyping was performed by real-time PCR amplification on an Applied Biosystems (ABI) 3500 apparatus using ABI TaqMan assays purchased from the manufacturer. The ABI assay identification numbers are given in Table 1.

### 2.4. Statistical Analysis

The frequency of alleles and genotypes were compared in Q1 and Q4 by the chi-square (*χ* ^2^) test. Bonferroni corrections to the significance of associations of the PGC1*α* Gly482Ser polymorphism with weight gain were not made because of our study design that placed *PPAR*γ**2 and *PGC1*α** as top priority candidate genes (Table 1). All of the genes we tested had already been reported to be associated with either T2DM or obesity.

## 3. Results

A total of 252 DCCT non-Hispanic white North American subjects (141 females and 111 males) comprising the upper and lower quartiles of weight gain with intensive therapy were initially genotyped, in a double-blinded manner, for a single common SNP in each of 7 candidate genes (Table 1). The subjects were originally stratified into quartiles regardless of gender. Quartile 1 (Q1) had 64 females and 63 males gained the least amount of weight and quartile 4 (Q4) had 85 females and 51 males and gained the highest amount of weight. Allelic and genotypic frequencies were initially compared between Q1 and Q4 regardless of gender. The results of this analysis revealed a significant difference in allelic and genotypic frequencies of the Gly482Ser polymorphism (rs8192678) in *PPARGC1A*. The Gly/Gly genotype frequency was 0.49 and 0.32 in Q1 and Q4, respectively, (*P* = .028) and the Gly/Ser genotype was 0.42 and 0.58 in Q1 and Q4, respectively, (*P* = .028). The genotypic distributions were in Hardy-Weinberg equilibrium (*P* = .25). The frequency of the Ser allele was 0.30 and 0.39 in Q1 and Q4, respectively, (*P* = .08). The frequencies of alleles and genotypes in all other 6 candidate genes were not significantly different between Q1 and Q4.

Next, we asked whether gender plays a role in the observed association. Interestingly, we found that the Gly482Ser polymorphism in *PPARGC1A* was associated with weight gain on intensive diabetes therapy only in males (Table 2). The frequency of the Ser allele in males of Q1 was 0.23 compared to 0.45 in males of Q4 (*P* = .005). The Ser allele frequency was almost identical in females of Q1 and Q4. As expected, the frequencies of genotypes in males were also significantly different between the two quartiles (Table 2). No other covariates were tested for association of the polymorphisms with weight gain.

**Figure 1:**
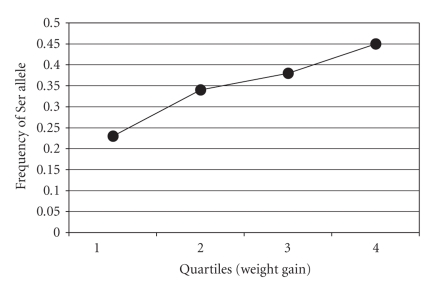
Association of the *PPARGC1A* Ser^482^ allele with weight gain in all quartiles of males with T1DM on intensive diabetes therapy. The frequency of the Ser allele in Q1 (*N* = 126) was 0.23; Q2 (*N* = 172) was 0.34; Q3 (*N* = 158) was 0.38; Q4 (*N* = 102) was 0.45. The Ser allele was significantly associated with a trend for higher weight gain with a *P*-value of .004 (Chi-square with 3 degrees of freedom), and a *P*-value of .0004 (trend, Cochran-Armitage).

In an attempt to validate the above association, we genotyped male subjects belonging to Q2 and Q3 for the Gly482Ser polymorphism and examined the association with the extent of weight gain on intensive diabetes therapy in all quartiles. The results are plotted in Figure 1. The frequency of the Ser allele in Q1 (*N* = 126) was 0.23; Q2 (*N* = 172) was 0.34; Q3 (*N* = 158) was 0.38; Q4 (*N* = 102) was 0.45. As observed with Q1 and Q4, the Ser allele was significantly associated with a trend for higher weight gain with a *P*-value of .004 (Chi-square with 3 degrees of freedom), and a *P*-value of .0004 (trend, Cochran-Armitage).

No significant differences in allele and genotype differences between Q1 and Q4 in either males or females were found for the rest of the candidate genes. The Gly482Ser polymorphism is widespread among various ethnic groups. The frequency of the Ser allele was reported to be 0.34 among Germans [10], 0.30 among Danes [8], 0.34 among European Americans (this study), 0.13 among African Americans (unpublished data from our laboratory), 0.18 among Pima Indians [29], 0.43 among Japanese [9], and 0.39 among Chinese (dbSNP at http://www.ncbi.nlm.nih.gov/SNP/).

**Figure 2:**
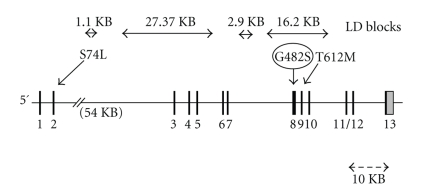
Structure of the *PPARGC1A* gene showing location of linkage disequilibrium (LD) blocks and sequence polymorphisms.

Subsequently, we assessed the association of a second polymorphism (rs 2279525) located in the 5′ untranslated region (5′ UTR) of the PGC-1*α* gene (Figure 2). No association with weight gain was observed in either males or females (Table 2). This polymorphism is approximately 46.6 KB upstream of the Gly482Ser polymorphism [30], and the two polymorphisms are in linkage equilibrium (http://www.hapmap.org/ and our data).

## 4. Discussion

We found that the common and widespread Gly482Ser polymorphism in PGC1*α* is significantly associated with excessive weight gain in male but not female subjects with T1DM who had undergone intensive diabetes therapy. The less common Ser allele was associated with a higher level of weight gain. Subjects who gained excessive weight had a higher frequency of T2DM among their parents. Therefore, we hypothesized that the Gly482Ser polymorphism may lead to excessive weight gain in DCCT via its association with the metabolic syndrome and T2DM.

PGC1*α* is a powerful transcriptional coactivator of several nuclear receptors, including PPAR*γ*, that regulate key metabolic steps in energy homeostasis and glucose metabolism. The level of PGC1*α* mRNA in skeletal muscle was observed to be lower in individuals with insulin resistance and T2DM [31]. In addition, the expression of PGC1*α*-responsive genes involved in oxidative phosphorylation is reduced in skeletal muscle of individuals with T2DM, their healthy first degree relatives and even those with impaired glucose tolerance [13, 14]. Importantly, PGC1*α* mRNA levels in skeletal muscle were lower among carriers of the Ser^482^ allele [31]. Therefore, it is likely that PGC1*α* with Ser at position 428 is a less efficient coactivator of transcription factors, including those that regulate the *PPARGC1A* gene itself [32]. Therefore, lower PGC1*α* activity levels may lead to lower levels of glucose and fatty acid oxidation and to fat accumulation [14].

In this study, the Ser^482^ allele predicted excessive weight gain with intensive diabetes therapy in males, but not in females. Two studies have also reported a gender-specific association of the Ser allele with obesity. Ridderstråle et al. reported association of the Ser allele with increased risk of obesity in physically inactive men over 50 years of age, but not in women [33]. However, in another study [10] the association was observed in middle-aged women but not men. The reason for this conflicting result could be attributed to the older age of subjects than those in DCCT, for example, the above middle-aged women could have been postmenopausal. Also, our subjects had T1DM and their weight gain was due specifically to intensive diabetes therapy. The Ser allele was also found to be associated with T2DM in Northern Chinese, especially in males [34]. We do not have an explanation for the gender-specific association. One possibility is that activity levels of PGC1*α* may be coregulated by estrogens. This is compatible with the observation that the level of muscle PGC1*α* mRNA is lower in females than males [31]. Therefore, effects of the Gly482Ser polymorphism on weight gain may be attenuated by the action of estrogens.

The following genetic association studies support the hypothesis that the PGC1*α* Gly482Ser polymorphism affects weight gain via its effect on the metabolic syndrome (which includes insulin resistance and abdominal obesity) and T2DM. First, a meta-analysis showed a modest association of the Ser^482^ allele with the risk for T2DM [35]. These case-control study populations included both males and females (3,718 cases and 4,818 controls) that were, unfortunately, not independently tested for association. Second, a locus on chromosome 4p15.1, where the *PPARGC1A* is located, was found to be associated with abdominal obesity [36] and insulin resistance [37]. Third, the PPAR*γ*2 Pro12Ala and the PGC1*α* Gly482Ser polymorphisms were associated with conversion from impaired glucose tolerance to T2DM in the STOP-NIDDM trial [38]. The PPAR*γ*2 Pro12Ala polymorphism has been consistently shown to be associated with risk for T2DM [6, 7, 39]. Fourth, The Gly482Ser polymorphism is located in a highly conserved domain that was shown to interact with and coactivate the muscle enhancer factor 2C that activates the GLUT4 gene [40]. Importantly, the Ser^482^ allele was consistently observed to be associated with insulin resistance, T2DM or obesity. This monoallelic association in different populations represents a strong validation for this association.

Haplotype analysis at the *PPARGC1A* gene locus in Caucasians revealed the existence of 4 adjacent multi-SNP linkage disequilibrium (LD) blocks with a D′ of close to 1 (Figure 2) (http://www.hapmap.org/). The Gly482Ser polymorphism lies within the 16.2 KB LD block. This block contains 10 haplotypes based on 8 single-SNP genotypes (http://www.broad.mit.edu/mpg/haploview/). Only one haplotype contains the Ser allele and is the most common one (0.367). The other seven haplotypes have Gly, the most common (0.224) differs from the Ser-containing haplotype at 8 of the 13 SNPs. Therefore, it is possible that variants in LD with the Ser allele could contribute to the observed association. Another polymorphism in exon 9 (Thr612Met) (Figure 2) that lies within the 16.2 KB LD block could also influence the activity of PGC1*α* [8]. However, its frequency is quite low in the general population (Met allele frequency of 0.03) and, therefore, is unlikely to have a major impact on the observed association with the Ser482 allele.

The polymorphism in the 5′ untranslated region of *PPARGC1A* (Figure 2) was not associated with weight gain in either sex in our study. Since this polymorphism is in LD with those in exon 1, our result is consistent with lack of association between the Ser74Leu polymorphism in exon 2 (Figure 2) and T2DM [8].

No significant association of variants in the other 7 candidate genes was observed. This does not mean that these genes do not contribute to excessive weight gain. The relatively small sample size and the low frequency of some of the polymorphisms may have resulted in low power to detect an association. A more thorough analysis of other variants in these genes is necessary using a larger population sample in order to detect moderate effects on weight gain.

## 5. Conclusions

The common PGC1*α*-Gly482Ser polymorphism was significantly associated with excessive weight gain in male, but not female subjects with T1DM who had undergone intensive diabetes therapy. The less common Ser allele was associated with a higher level of weight gain. PGC1*α* Ser is a less efficient coactivator of transcription factors. Therefore, lower PGC1*α* activity levels may lead to lower levels of glucose and fatty acid oxidation and to fat accumulation.

## Figures and Tables

**Table 1:** *Candidate genes and SNPs analyzed for association with excess weight gain with intensive diabetes therapy.* Genotyping was performed using ABI TaqMan assays with the indicated ABI identification numbers (custom, made to order). Minor allele frequencies are for Caucasian populations derived from published data and the SNP database (http://www.ncbi.nlm.nih.gov/SNP/). *PPARG*, peroxisome proliferator activated receptor *γ*; *PPARGC1A*, peroxisome proliferator-activated receptor *γ* coactivator-1*α*; *HSD11B1*, 11*β*-hydroxysteroid dehydrogenase 1; GR (*NRC31*), glucocorticoid receptor; *GNB3,* G protein *β*3 subunit; *ADIPOQ*, adiponectin; *NPY*, neuropeptide y, *FTO* (aliases *KIAA1752*, *MGC5149*), fat mass, and obesity-associated protein.

Gene	Chromosomal location	NCBI refSNP number	TaqMan ABI assay ID	Polymorphism	Minor allele frequency
*PPAR*γ*2*	3p25	rs1801282	1129864	Pro12Ala (exon 1)	0.10
*PPARGC1A*	4p15.1	rs8192678	Custom 1643184	Gly482Ser T/C (5′ UTR)	0.34
		rs2279525			0.31
*11-*β* HSD1*	1q32	rs2884090	2502457	C/T; intron 4	0.21
*GR (NRC31)*	5q34	rs6188	1046353	G/T; intron 5	0.31
		rs11749561	178285	T/C; intron 3	
*GNB3*	12p13	rs5443	2184734	C825T Silent/AS	0.30
*ADIPOQ*	3q27	rs266729	Custom 2412786	C-377G; promoter	0.29
*NPY*	7p15.1	rs5573	11164468	Ser22Ser	0.44
*FTO*	16q12.2	rs9939609	Custom 30090620	T to A in 3′ UTR	0.43

**Table 2:** *Genotype and allele frequencies of the variants in candidate genes examined for association with weight gain by DCCT subjects on intensive diabetes therapy.* *N*, number of subjects; the *P*-values compare genotype and minor allele frequencies between quartile 1 (Q1) and quartile 4 (Q4) of weight gain; NS, statistically not significant. The brackets indicate that heterozygotes and homozygotes for the minor allele were combined for comparisons between quartiles.

Gene/polymorphism	Frequency in females			Frequency in males		
	Q1 (*N*, %)	Q4 (*N*, %)	*P*	Q1 (*N*, %)	Q4 (*N*, %)	*P*
*PPARG2*						
* * * * * * *Pro/Pro *	52 (82.5)	62 (78.5)	NS	45 (75.0)	42 (84.2)	NS
* * * * * * *Pro/Ala*	11 (17.5)	17 (21.5)	NS	15 (25.0)	9 (17.6)	NS
* * * * * * *Ala/Ala *	0 (0)	0 (0)		0 (0)	0 (0)	NS
Allele frequency	0.09	0.11	NS	0.13	0.09	NS

* PPARGC1A*						
* * * * * * *Gly/Gly *	25 (40.3)	30 (38.0)	NS	35 (58.3)	12 (23.5)	.0054
* * * * * * *Gly/Ser*	$[\begin{array}{c} 28\;\;(45.2)\\ 9\;\;(14.5) \end{array}$	$\begin{array}{c} 43\;\;(54.4)\\ 6\;\;(7.6) \end{array}]$	NS	$[\begin{array}{c} 22\;\;(36.7)\\ 3\;\;(5.0) \end{array}$	$\begin{array}{c} 32\;\;(62.8)\\ 7\;\;(13.7) \end{array}]$	.0131
* * * * * * *Ser/Ser*						
Frequency of Ser	0.37	0.35	NS	0.23	0.45	.0050

*PPARGC1A*						
* * * * * * $T/\overset{˜}{T}$	28 (45.2)	35 (44.9)	NS	25 (42.3)	28 (54.9)	NS
* * * * * * $T/\overset{˜}{C}$	$[\begin{array}{c} 27\;\;(43.5)\\ 7\;\;(11.3) \end{array}$	$\begin{array}{c} 38\;\;(48.7)\\ 5\;\;(6.4) \end{array}]$	NS	$[\begin{array}{c} 26\;\;(44.1)\\ 8\;\;(13.6) \end{array}$	$\begin{array}{c} 19\;\;(37.3)\\ 4\;\;(7.8) \end{array}]$	NS
* * * * * * *C/C*						
Frequency of *C*	0.33	0.30	NS	0.35	0.27	NS

*HSD11B1*						
* * * * * * *C/C*	41 (66.1)	50 (59.5)	NS	42 (66.7)	32 (62.7)	NS
* * * * * * *C/T*	$[\begin{array}{c} 18\;\;(29.0)\\ 3\;\;(4.9) \end{array}$	$\begin{array}{c} 28\;\;(33.3)\\ 6\;\;(7.2) \end{array}]$	NS	$[\begin{array}{c} 16\;\;(25.4)\\ 5\;\;(7.9) \end{array}$	$\begin{array}{c} 17\;\;(33.3)\\ 2\;\;(4.0) \end{array}]$	NS
* * * * * * *T/T*						
Frequency of *T*	0.19	0.23	NS	0.21	0.21	NS

*GNB3*						
* * * * * * *C/C*	34 (54.8)	35 (44.3)	NS	30 (50.0)	28 (54.9)	NS
* * * * * * *C/T*	$[\begin{array}{c} 23\;\;(37.1)\\ 5\;\;(8.1) \end{array}$	$\begin{array}{c} 33\;\;(41.8)\\ 11\;\;(13.9) \end{array}]$	NS	$[\begin{array}{c} 23\;\;(38.3)\\ 7\;\;(11.7) \end{array}$	$\begin{array}{c} 19\;\;(37.3)\\ 4\;\;(7.8) \end{array}]$	NS
* * * * * * *T/T*						
Frequency of *T*	0.27	0.35	NS	0.31	0.27	NS

*NRC31*						
* * * * * * *G/G*	27 (42.2)	41 (52.6)	NS	29 (48.3)	25 (49.0)	NS
* * * * * * *G/T*	$[\begin{array}{c} 29\;\;(45.3)\\ 8\;\;(12.5) \end{array}$	$\begin{array}{c} 34\;\;(43.6)\\ 3\;\;(3.8) \end{array}]$	NS	$[\begin{array}{c} 22\;\;(36.7)\\ 9\;\;(15.0) \end{array}$	$\begin{array}{c} 25\;\;(49.0)\\ 1\;\;(2.0) \end{array}]$	NS
* * * * * * *T/T*						
Frequency of *T*	0.35	0.26	NS	0.33	0.27	NS

*ADIPOQ *						
* * * * * * *C/C*	32 (52.5)	49 (62.0)	NS	32 (53.3)	30 (58.8)	NS
* * * * * * *C/G *	$[\begin{array}{c} 22\;\;(36.1)\\ 7\;\;(11.4) \end{array}$	$\begin{array}{c} 24\;\;(30.4)\\ 6\;\;(7.6) \end{array}]$	NS	$[\begin{array}{c} 26\;\;(43.4)\\ 2\;\;(3.3) \end{array}$	$\begin{array}{c} 17\;\;(33.3)\\ 4\;\;(7.9) \end{array}]$	NS
* * * * * * *G/G*						
Frequency of *G*	0.30	0.23	NS	0.25	0.25	NS

*NPY*						
* * * * * * *G/G*	19 (30.6)	21 (26.6)	NS	15 (25.0)	10 (19.6)	NS
* * * * * * *G/A*	$[\begin{array}{c} 29\;\;(46.8)\\ 14\;\;(22.6) \end{array}$	$\begin{array}{c} 34\;\;(43.0)\\ 24\;\;(30.4) \end{array}]$	NS	$[\begin{array}{c} 32\;\;(53.3)\\ 13\;\;(21.7) \end{array}$	$\begin{array}{c} 27\;\;(52.9)\\ 14\;\;(27.5) \end{array}]$	NS
* * * * * * *A/A*						
Frequency of *A*	0.46	0.52	NS	0.48	0.54	NS

FTO						
* * * * * * *TT*	23 (36.0)	29 (35.0)	NS	16 (25.4)	13 (27.1)	NS
* * * * * * *TA*	32 (50.0)	40 (48.2)	NS	35 (55.6)	25 (52.1)	NS
* * * * * * *AA*	9 (14.0)	14 (17.0)	NS	12 (19.0)	10 (20.8)	NS
Frequency of *A*	0.39	0.41	NS	0.47	0.47	NS

## References

[1] Krolewski AS, Kosinski EJ, Warram JH (1987). Magnitude and determinants of coronary artery disease in juvenile-onset, insulin-dependent diabetes mellitus. *The American Journal of Cardiology*.

[2] The Diabetes Control and Complications Trial Research Group (1993). The effect of intensive treatment of diabetes on the development and progression of long-term complications in insulin-dependent diabetes mellitus. *The New England Journal of Medicine*.

[3] Purnell JQ, Hokanson JE, Marcovina SM, Steffes MW, Cleary PA, Brunzell JD (1998). Effect of excessive weight gain with intensive therapy of type 1 diabetes on lipid levels and blood pressure: results from the DCCT. *The Journal of the American Medical Association*.

[4] Purnell JQ, Dev RK, Steffes MW (2003). Relationship of family history of type 2 diabetes, hypoglycemia, and autoantibodies to weight gain and lipids with intensive and conventional therapy in the diabetes control and complications trial. *Diabetes*.

[5] Carr MC, Brunzell JD (2004). Abdominal obesity and dyslipidemia in the metabolic syndrome: importance of type 2 diabetes and familial combined hyperlipidemia in coronary artery disease risk. *The Journal of Clinical Endocrinology & Metabolism*.

[6] Deeb SS, Fajas L, Nemoto M (1998). A Pro12Ala substitution in PPAR*γ*2 associated with decreased receptor activity, lower body mass index and improved insulin sensitivity. *Nature Genetics*.

[7] Altshuler D, Hirschhorn JN, Klannemark M (2000). The common PPAR*γ*
Pro12Ala polymorphism is associated with decreased risk of type 2 diabetes. *Nature Genetics*.

[8] Ek J, Andersen G, Urhammer SA (2001). Mutation analysis of peroxisome proliferator-activated receptor-*γ*
coactivator-1 (PGC-1) and relationships of identified amino acid polymorphisms to type II diabetes mellitus. *Diabetologia*.

[9] Hara K, Tobe K, Okada T (2002). A genetic variation in the PGC-1 gene could confer insulin resistance and susceptibility to type II diabetes. *Diabetologia*.

[10] Esterbauer H, Oberkofler H, Linnemayr V (2002). Peroxisome proliferator-activated receptor-*γ* coactivator-1 gene locus: associations with obesity indices in middle-aged women. *Diabetes*.

[11] Puigserver P, Spiegelman BM (2003). Peroxisome proliferator-activated receptor-*γ* coactivator 1*α* (PGC-1*α*): transcriptional coactivator and metabolic regulator. *Endocrine Reviews*.

[12] Shuldiner AR, McLenithan JC (2004). Genes and pathophysiology of type 2 diabetes: more than just the Radle cycle all over again. *The Journal of Clinical Investigation*.

[13] Mootha VK, Lindgren CM, Eriksson K-F (2003). PGC-1*α*-responsive genes involved in oxidative phosphorylation are coordinately downregulated in human diabetes. *Nature Genetics*.

[14] Patti ME, Butte AJ, Cusi K (2003). Coordinated reduction of genes of oxidative metabolism in humans with insulin resistance and diabetes: potential role of *PGC1* and *NRF1*. *Proceedings of the National Academy of Sciences of the United States of America*.

[15] Dobson MG, Redfern CPF, Unwin N, Weaver JU (2001). The N363S polymorphism of the glucocorticoid receptor: potential contribution to central obesity in men and lack of association with other risk factors for coronary heart disease and diabetes mellitus. *The Journal of Clinical Endocrinology & Metabolism*.

[16] Rosmond R, Chagnon YC, Holm G (2000). A glucocorticoid receptor gene marker is associated with abdominal obesity, leptin, and dysregulation of the hypothalamic-pituitary-adrenal axis. *Obesity Research*.

[17] van Rossum EFC, Lamberts SWJ (2004). Polymorphisms in the glucocorticoid receptor gene and their associations with metabolic parameters and body composition. *Recent Progress in Hormone Research*.

[18] Rask E, Olsson T, Soderberg S (2001). Tissue-specific dysregulation of cortisol metabolism in human obesity. *The Journal of Clinical Endocrinology & Metabolism*.

[19] Masuzaki H, Paterson J, Shinyama H (2001). A transgenic model of visceral obesity and the metabolic syndrome. *Science*.

[20] Seckl JR, Walker BR (2001). Minireview: 11*β*-hydroxysteroid dehydrogenase type 1—a tissue-specific amplifier of glucocorticoid action. *Endocrinology*.

[21] Siffert W (2000). G protein 
*β*
_3_ subunit 825T allele, hypertension, obesity, and diabetic nepropathy. *Nephrology Dialysis Transplantation*.

[22] Rydén M, Faulds G, Hoffstedt J, Wennlund A, Arner P (2002). Effect of the (C825T) G*β*
_3_ polymorphism on adrenoceptor-mediated lipolysis in human fat cells. *Diabetes*.

[23] Stefan N, Stumvoll M, Machicao F, Koch M, Häring HU, Fritsche A (2004). C825T polymorphism of the G protein 
*β*
_3_ subunit is associated with obesity but not with insulin sensitivity. *Obesity Research*.

[24] Kissebah AH, Sonnenberg GE, Myklebust J (2000). Quantitative trait loci on chromosomes 3 and 17 influence phenotypes of the metabolic syndrome. *Proceedings of the National Academy of Sciences of the United States of America*.

[25] Menzaghi C, Ercolino T, Paola RD (2002). A haplotype at the adiponectin locus is associated with obesity and other features of the insulin resistance syndrome. *Diabetes*.

[26] Hara K, Boutin P, Mori Y (2002). Genetic variation in the gene encoding adiponectin is associated with an increased risk of type 2 diabetes in the Japanese population. *Diabetes*.

[27] Frayling TM, Timpson NJ, Weedon MN (2007). A common variant in the *FTO* gene is associated with body mass index and predisposes to childhood and adult obesity. *Science*.

[28] Dina C (2008). New insights into the genetics of body weight. *Current Opinion in Clinical Nutrition and Metabolic Care*.

[29] Muller YL, Bogardus C, Pedersen O, Baier L (2003). A Gly482Ser missense mutation in the peroxisome proliferator-activated receptor *γ*
coactivator-1 is associated with altered lipid oxidation and early insulin secretion in Pima Indians. *Diabetes*.

[30] Esterbauer H, Oberkofler H, Krempler F, Patsch W (1999). Human peroxisome proliferator activated receptor gamma coactivator 1 (*PPARGC1*) gene: cDNA sequence, genomic organization, chromosomal localization, and tissue expression. *Genomics*.

[31] Ling C, Poulsen P, Carlsson E (2004). Multiple environmental and genetic factors influence skeletal muscle *PGC-1α*
and *PGC-1β* gene expression in twins. *The Journal of Clinical Investigation*.

[32] Handschin C, Rhee J, Lin J, Tarr PT, Spiegelman BM (2003). An autoregulatory loop controls peroxisome proliferator-activated receptor *γ*
coactivator 1*α*
expression in muscle. *Proceedings of the National Academy of Sciences of the United States of America*.

[33] Ridderstråle M, Johansson LE, Rastam L, Lindblad U (2006). Increased risk of obesity associated with the variant allele of the PPARGC1A Gly482Ser polymorphism in physically inactive elderly men. *Diabetologia*.

[34] Sun L, Yang Z, Jin F (2006). The Gly482Ser variant of the PPARGC1 gene is associated with type 2 diabetes mellitus in northern Chinese, especially men. *Diabetic Medicine*.

[35] Barroso I, Luan J, Sandhu MS (2006). Meta-analysis of the Gly482Ser variant in *PPARGC1A* in type 2 diabetes and related phenotypes. *Diabetologia*.

[36] Pérusse L, Rice T, Chagnon YC (2001). A genome-wide scan for abdominal fat assessed by computed tomography in the Québec Family Study. *Diabetes*.

[37] Pratley RE, Thompson DB, Prochazka M (1998). An autosomal genomic scan for loci linked to prediabetic phenotypes in pima indians. *The Journal of Clinical Investigation*.

[38] Andrulionytè L, Zacharova J, Chiasson J-L, Laakso M (2004). Common polymorphisms of the *PPAR*-*γ*2 (*Pro12Ala*) and *PGC-1α*
(*Gly482Ser*) genes are associated with the conversion from impaired glucose tolerance to type 2 diabetes in the STOP-NIDDM trial. *Diabetologia*.

[39] Scott LJ, Mohlke KL, Bonnycastle LL (2007). A genome-wide association study of type 2 diabetes in finns detects multiple susceptibility variants. *Science*.

[40] Michael LF, Wu Z, Cheatham RB (2001). Restoration of insulin-sensitive glucose transporter (GLUT_4_) gene expression in muscle cells by the transcriptional coactivator PGC-1. *Proceedings of the National Academy of Sciences of the United States of America*.

